# A case of Sjögren's syndrome with worsening of pleural effusion due to steroid discontinuation

**DOI:** 10.1016/j.rmcr.2021.101391

**Published:** 2021-03-19

**Authors:** Osamu Usami

**Affiliations:** Kurihara Central Hospital, Department of Respiratory Medicine, Miyano Cyuo 3-11, Tsukidate, Kurihara, Miyagi, 987-2205, Japan

**Keywords:** Anti-SS-A antibody, Pleural effusion, Sjögren's syndrome, Steroids

## Abstract

The etiology of Sjögren's syndrome (SS) remains unclear and is associated with many other autoimmune diseases. In particular, symptoms of SS are not apparent when steroids are already being administered for other autoimmune diseases. This report documents a case of autoimmune hepatitis with SS, which was diagnosed on the basis of the worsening of unilateral pleural effusion after the discontinuation of steroids as well as the manifestation of symptoms, such as dry mouth. Adrenal insufficiency due to the discontinuation of steroids was assumed to be the cause of the worsening, rather than infection stress, because no indicators of infectious diseases were observed. The diagnosis of SS was confirmed via lip biopsy examination and anti-SS antibody positivity. Re-administration of steroids rather than antibiotics drastically reduced the pleural effusion and improved the dry mouth symptom. SS with pleural effusion in a case of autoimmune disease was reported to show both unilateral and bilateral pleural effusion predominantly containing lymphocytes. SS with pleural effusion may be more common than expected and should be differentiated from traditional SS. Moreover, biopsy examination should be considered if necessary because the condition might remain latent when steroids are administered.

## Highlights

• Sjögren's syndrome might be masked by steroid treatment for other conditions.
• Pleural effusion after steroid discontinuation might indicate Sjögren's syndrome.
• Lip biopsy examination and anti-SS antibody tests help confirm Sjögren's syndrome.
• Re-administration of steroids helps reverse the condition and improve symptoms.

## 1 Background

Sjögren's syndrome (SS) is an autoimmune disease. Its main characteristic physical findings are dry mouth and dry eye. Although the etiology of SS remains unclear, factors such as genetic predisposition (human leukocyte antigen), endocrine abnormalities, immune disorders, and infectious diseases are known to lead to its onset [1,2]. The main pulmonary complication of SS is pulmonary fibrosis. Cases of unilateral pleural effusion in SS are relatively rare. Moreover, pleural effusion is often difficult to distinguish between cases of infectious and autoimmune diseases. This report documents a case of SS with a worsening of unilateral pleural effusion due to the discontinuation of steroids. The patient did not show any infectious symptoms, and the symptoms improved after the re-administration of steroids.

## 2 Case presentation

**Fig. 1:**
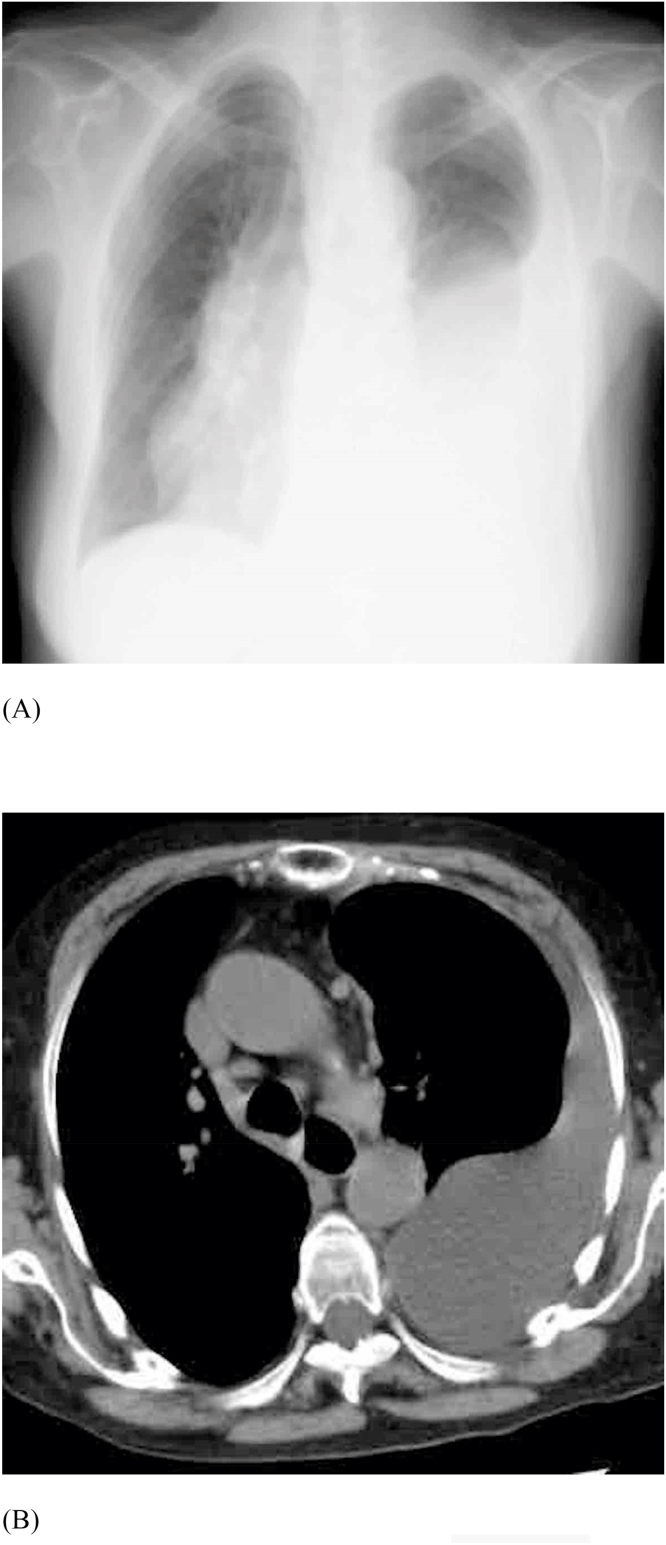
Chest radiogram (A) and computed tomography image (B) show left pleural effusion and mild lymphadenopathy. No obvious infiltrative shadows or interstitial shadows are seen in the lung field.

An 81-year-old woman who was taking oral prednisolone 5 mg per day for autoimmune hepatitis was referred to us with shortness of breath. Oxygen saturation was 94% at an oxygen flow rate of 4 L/min. She had clear consciousness, no erythema on the skin, no palpable body surface lymph nodes, and no cough. A sputum analysis revealed only indigenous bacteria. Two blood culture tests yielded negative results. The breath sounds were slightly diminished on the left side, but no abnormal sound was noted. The number of breaths was 12 per minute, and the body temperature was 36.4 °C. She did not complain of dry mouth or dry eye on admission. Her laboratory findings on admission showed normal white blood cell count and C-reactive protein and procalcitonin levels. Chest radiography revealed a blunt left rib diaphragm angle, suggesting pleural effusion on the left side (Fig. 1). Chest contrast-enhanced computed tomography revealed pleural effusion only on the left side. No abnormal findings were observed in the lung field. However, mild swelling of the mediastinal lymph nodes was noted. Although gallstones were present, obvious cirrhosis was not evident. A small amount of pleural effusion fluid was sampled via a puncture for analysis. The fluid appeared slightly cloudy and yielded a positive result on the Rivalta reaction. The specific gravity of the fluid was 1.037, lactate dehydrogenase level was 483 IU/L, protein level was 5.4 g/dL, adenosine deaminase (ADA) level was 37 IU/L, and glucose level was 89 mg/dL. Bacterial and mycobacterial cultures yielded negative results, and no malignant cells were observed. Many lymphocytes, some histiocytes, and a few mesothelial cells were also observed. The percentage of differential cells and hyaluronic acid level could not be measured at our hospital. The pleural fluid was considered exudative based on Light's criteria.

After admission, the patient showed no findings suggestive of an infection. However, because opportunistic infections could not be ruled out, the previously prescribed oral prednisolone 5 mg per day was discontinued. Although prednisolone discontinuation could cause adrenal insufficiency, the benefits of discontinuation were considered to be superior. However, her symptoms did not improve, and she developed fever and malaise. Despite the lack of findings suggestive of an infection, she was started on sulbactam/ampicillin for diagnostic treatment. Nevertheless, little improvement in oxygenation was observed. Moreover, she began to complain of a dry mouth. Her blood pressure also decreased, and so the amount of infusion was increased to support the low blood pressure.

**Fig. 2:**
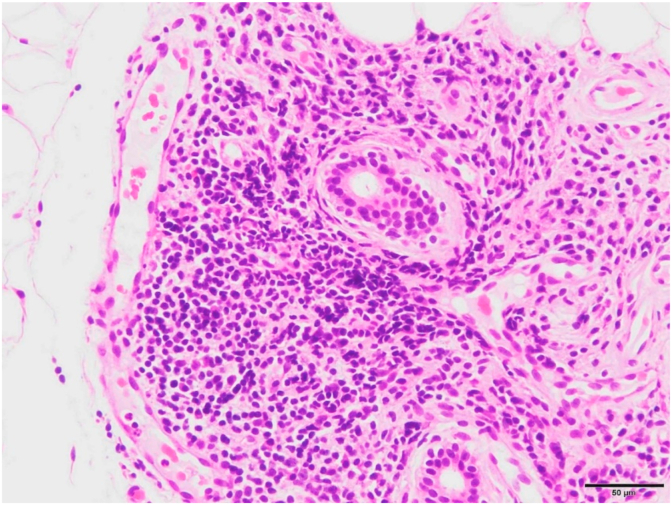
Lip biopsy specimen (hematoxylin and eosin staining, ×100) demonstrates lymphocyte infiltration into the minor salivary glands or lacrimal glands.

Subsequently, a blood examination revealed that she was positive for anti-SS-A, SS-B, and anti-ds-DNA antibodies. Since autoimmune hepatitis is frequently associated with autoimmune diseases and complaints of dry mouth, SS was suspected as the cause of the pleural effusion, and hence a lip biopsy examination was performed (Fig. 2). The examination revealed that the minor salivary glands with adipose tissue were slightly atrophied and infiltrated the layers around the conduit at 3–4 locations. No peculiar inflammatory symptoms such as granulomas were observed. Therefore, it histologically satisfied the diagnostic criteria for SS as chronic salivary gland inflammation. The Schirmer test results were also negative.

**Fig. 3:**
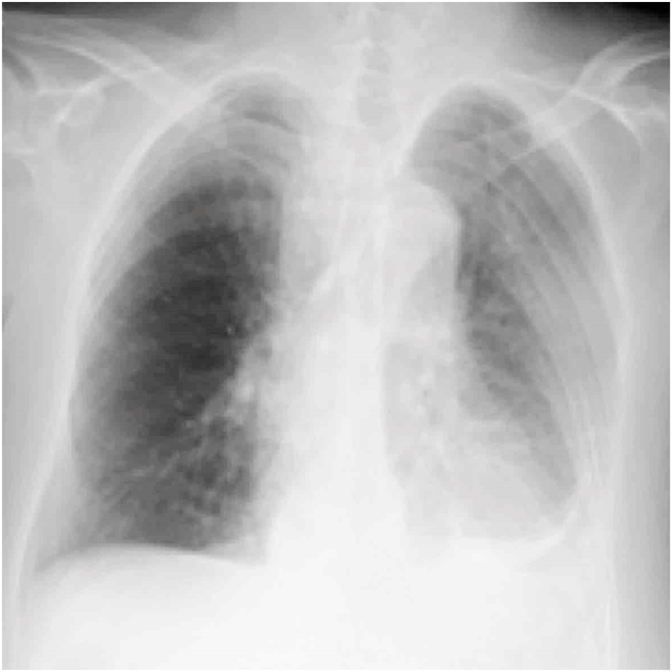
Left pleural effusion decreases after the re-administration of steroids.

Oral prednisolone30 mg per day was started to treat the suspected adrenal insufficiency and SS. Oxygenation improved 5 days after re-starting prednisolone administration. After the blood pressure recovered to normal, the left pleural effusion decreased (Fig. 3) and the dry mouth symptoms improved. The prednisolone dose was reduced to 20 mg per day, and the patient was discharged. Prednisolone administration was continued on an outpatient basis. She has since been recurrence free. The patient has provided consent for the publication of the case findings.

## 3 Discussion

SS is an autoimmune disease in which lymphocytes infiltrate exocrine glands, such as the salivary and lacrimal glands, and damage the glandular tissues. The most specific physical findings are dry mouth and dry eye [3]. Histopathological findings include lymphocyte infiltration around the intralobular conduit [4]. SS is classified as either primary SS, wherein the clinical manifestations occur alone, or secondary SS, which is associated with collagen diseases such as rheumatoid arthritis and systemic lupus erythematosus (SLE) [5]. Primary SS is further classified into the glandular type, wherein the lesion is localized to exocrine glands such as the lacrimal and salivary glands and exhibits only dry symptoms, and the extraglandular type, wherein the lesion extends to systemic organs other than the exocrine glands. Cases that do not show dry symptoms but show positive findings on an oral examination, ophthalmic examination, and blood tests are said to have latent SS and are distinguished from those that have overt SS with dry symptoms. The present case was diagnosed as overt SS on the basis of biopsy histopathological examination, SS-A and SS-B antibody positivity, and dry mouth. The general diagnostic criteria for SS are as follows: (1) biopsy histopathological examination (infiltration of 50 or more lymphocytes around the duct in the labial gland tissue or lacrimal gland tissue); (2) stage I or decreased salivary secretion detected using salivary gland imaging; (3) positive Schirmer test results; and (4) anti-Ro/SS-A antibody or anti-La/SS-B antibody positivity [6]. The present case was diagnosed as SS because it satisfied criteria 1 and 4. In addition, on the European League Against Rheumatism SS disease activity index, it received at least 5 points and became severe [7,8].

The patient also tested positive for anti-ds-DNA antibody and had pleural effusion, which met one of the diagnostic criteria for SLE [9]. However, she showed no SLE symptoms, such as butterfly erythema and lymphocyte depletion. Therefore, no definitive SLE diagnosis had yet been made. Her condition could not be confirmed as secondary SS, and it was speculated that it was latent primary SS. The patient was taking prednisolone for the previously diagnosed autoimmune hepatitis; this treatment presumably also suppressed the SS symptoms. However, as SS progressed, the steroid dosage was no longer sufficient to suppress the symptoms. Moreover, the discontinuation of the oral steroids after admission worsened the condition, causing the manifestation of SS symptoms. The decreased blood pressure was likely due to an adrenal crisis. Although the previous diagnostic process in this case was unknown, the diagnostic criteria of the autoimmune hepatitis clinical practice guidelines suggested it was a typical case of type 1 autoimmune hepatitis.

The most common causes of pleural effusion include lung cancer dissemination, metastatic tumors, tuberculous pleural inflammation, and malignant mesothelioma. However, in this case, although the pleural effusion was exudative, malignant cells were not identified, and empyema was absent. The ADA level was high, but tuberculosis was absent. ADA level in the pleural effusion fluid has excellent sensitivity and specificity for the diagnosis of tuberculous pleural inflammation, but it is also known to show nonspecific elevation in various diseases, including autoimmune diseases. Therefore, diagnosing tuberculosis on the basis of the pleural effusion ADA level alone is dangerous [10].

In the present case, the patient had SS that caused unilateral pleural effusion, which was accidentally discovered after steroid discontinuation. Unilateral pleural effusion should be cited as a possible differential diagnosis of collagen disease, and hence, steroids should not be readily discontinued. Moreover, pleural effusion associated with autoimmune diseases is unilateral in half of the reports, and this point should be considered during the differential diagnosis. SS is often associated with peripheral airway lesions and interstitial lung disease, and it is rare that pleural effusion is complicated in the lung field without shadows, especially in primary SS [[11], [12], [13]]. Most cases also show lymphocyte-dominant exudative pleural effusions, as did the present case [[14], [15], [16], [17]]. SS with pleural effusion is known to respond well to steroids, and this was also confirmed in the present case. However, established findings regarding the pathophysiology of and prognosis prediction in SS are currently lacking. Although studies suggest that human leukocyte antigen and viral infections are involved in the onset of SS, the present case showed no signs of infection.

To conclude, this case report highlights the importance of including Sjögren's syndrome as a possible differential diagnosis in patients who develop unilateral pleural effusion after the discontinuation of steroid therapy initiated for other autoimmune diseases.

## Funding

This research did not receive any specific grant from funding agencies in the public, commercial, or not-for-profit sectors.

## Declaration of competing interest

None.
